# An Adolescent Female With Disordered Eating and Cannabis Use Found to Have Acute Intermittent Porphyria

**DOI:** 10.1155/crps/8875138

**Published:** 2025-06-25

**Authors:** Brooke Gertz, Mark Mullen, Tony Pesavento

**Affiliations:** ^1^Department of Psychiatry, Creighton University School of Medicine, Omaha, Nebraska, USA; ^2^Department of Psychiatry, Saint Louis University School of Medicine, St. Louis, Missouri, USA; ^3^Department of Behavioral Health, Children's Nebraska Hospital and Medical Center, Omaha, Nebraska, USA

## Abstract

**Background:** Eating disorders and cannabinoid hyperemesis syndrome are increasingly common causes of nausea, vomiting, and weight loss in adolescent females. Acute intermittent porphyria (AIP) is rare but has considerable pathophysiological overlap with these conditions and requires a high index of suspicion.

**Purpose and Basic Procedures:** We present the case of a 15-year-old girl who presented with nausea, vomiting, and decreased appetite in the context of cannabis use and disordered eating. She was initially discharged from the emergency department but returned the next day experiencing seizures and altered mental status. Medical workup revealed AIP, and she responded well to the appropriate treatment.

**Main Findings:** To date, no literature exists about the overlap between cannabinoid hyperemesis syndrome and AIP, although they often present with similar features. There is scant information about the interplay between AIP and disordered eating. As our case report shows, an AIP diagnosis could be delayed by misattribution of presenting symptoms to cannabis use or disordered eating.

**Principal Conclusion:** AIP is a rare but highly treatable cause of nausea, vomiting, and altered mental status in adolescents. Due to its symptomatologic overlap with more common conditions like cannabinoid hyperemesis syndrome and eating disorders, it is easily missed. Thus, a high index of suspicion is required to obtain an AIP diagnosis and initiate treatment.

## 1. Introduction

The causes of recurrent nausea, vomiting, weight loss, and decreased appetite in an adolescent are numerous. An increasingly common etiologic consideration for persistent nausea/vomiting not related to viral illness is cannabinoid hyperemesis syndrome, characterized by cyclical abdominal pain, nausea, and vomiting that occur every few weeks to months in the context of chronic cannabis use [1]. The symptoms resolve after sustained discontinuation of cannabis use. Also high on the differential are primary psychiatric considerations such as eating disorders. Eating disorders are relatively common with a 12-month prevalence of 2.1% in women, and adolescent women are at particularly high risk [2]. A less common diagnostic consideration is acute intermittent porphyria (AIP), a metabolic disorder of dysfunctional heme synthesis, which most frequently presents with abdominal pain but can manifest in a variety of neuropsychiatric symptoms ranging from peripheral neuropathy and tachycardia to delirium and hallucinations. Acute attacks persist from days to weeks, and diagnosis is often delayed due to nonspecific symptoms [3]. AIP overlaps physiologically with eating disorders since carbohydrate restriction is a precipitant for attacks, and attacks lead to lack of appetite, emesis, and weight loss. We report the case of a girl with nausea, vomiting, and a history of chronic cannabis use and an eating disorder in which her AIP may easily have been misdiagnosed or dismissed due to her history.

AIP involving nausea and vomiting in an adolescent with a history of chronic cannabis use and an eating disorder who may easily have been misdiagnosed or dismissed due to her history. We discuss the workup for AIP, including a broad range of differential diagnoses, as well as diagnostic recommendations to guide clinicians toward AIP testing when it is indicated.

## 2. Case Report

A 15-year-old girl was brought to the emergency department by her parents after 6 days of abdominal pain and vomiting. Symptoms began abruptly and consisted of multiple episodes of emesis per day, with difficulty keeping down any food. The patient did not have any bowel movements since the onset of symptoms. Abdominal ultrasound ruled-out bowel obstruction. Her parents reported that in addition to intentional weight loss over the past year, the patient unintentionally lost eight pounds (lbs; 3.63 kg) over the past week. Her abdomen was diffusely tender to palpation with hyperactive bowel sounds. Vitals were notable for elevated blood pressure, with systolic readings of about 130 mm Hg. Basic laboratory investigations revealed mildly elevated hemoglobin (11 g/dL, range 12.1–15.6), mild hyponatremia (131 mmol/L, range 135–147), mildly elevated liver enzymes (ALT 40 U/L, range 10–35; AST 43 U/L, range 10–40), and the presence of urine ketones. Renal function was normal. Lipase was normal, which decreased concern for pancreatitis. The urine drug screen was positive for cannabinoids, and the patient endorsed use every few days for the past 4 months. The patient also noted that hot showers had been helpful in improving her symptoms, which is common in cannabinoid hyperemesis syndrome but not specific to this disorder [4]. The emergency department note documented suspicion for viral gastroenteritis versus cannabinoid hyperemesis syndrome and that the patient was discharged home with ondansetron. Ondansetron had not provided symptomatic relief, and the patient used cannabis after coming home from the emergency department. She returned the next day with her parents reporting seizure-like activity (arm and leg stiffening, fixed gaze) followed by confusion and altered mental status with agitation that required 2 mg haloperidol, 1 mg lorazepam, and 25 mg diphenhydramine in the emergency department.

At that time, electroencephalogram (EEG) revealed bilateral posterior slowing, and magnetic resonance imaging (MRI) showed bilateral, symmetric cortical signal within the frontal, parietal, and occipital lobes. The patient received a loading dose of levetiracetam as well as a methylprednisolone burst for possible autoimmune encephalitis based on EEG and MRI results.

Our psychiatry team was consulted on hospital day 2 and noted symptoms of encephalopathy to include delayed and non-sequitur responses and intermittent disorientation. We discovered that the patient had a history of restricted caloric intake and purging about 1 year ago and had recently been restricting caloric intake, which she attributed to nausea. Per family, the patient had lost 35 lbs (15.9 kg) from her maximum weight, and her pediatrician reported a 65 lb (29.5 kg) weight loss over the past 2 years. Body mass index on admission was 16.79 kg/m^2^. Per documentation, the pediatrician had expressed concern for an eating disorder, and the patient had received some outpatient treatment for disordered eating several years ago. The patient's mother explained that the patient would not eat lunch at school the previous year, and the patient reported that her food intake consisted of periodic snacking rather than meals. When the patient was asked to provide an example of her daily diet, she struggled to list specific foods. Her mother added that they always had healthy snacks available for the patient to eat, such as apples, salads, and vegetables. Although the patient currently denied any desire to lose weight, she reported that she wanted to lose weight the previous year. The patient reported that it had been more than 1 year since she last self-induced vomiting. The patient denied excessive exercise, and her mother reported that she had encouraged the patient to start an exercise regimen to be more active. The patient reported that she was having regular monthly menstrual cycles, and she was not using any oral contraceptive.

Further medical analysis revealed normal tissue transglutaminase immunoglobulin A antibody (obtained in consideration of celiac disease given nausea/vomiting), normal aldosterone and renin activity (obtained to investigate hypertension), and normal iron and total iron binding capacity (obtained in consideration of hemochromatosis given abdominal pain, elevated liver enzymes, and elevated hemoglobin). Ferritin was elevated to 189 ng/mL (range 10–70) and C-reactive protein was elevated to 0.8 mg/dL (range 0–0.7), both of which may represent an inflammatory process. The patient was negative for syphilis and human immunodeficiency virus (obtained in consideration of an infectious cause of encephalopathy) and chlamydia and gonorrhea (obtained in consideration of pelvic inflammatory disease, though not of high concern as a primary differential diagnosis). The following tests were also negative: antineutrophilic cytoplasmic antibody-associated vasculitis profile (obtained in consideration of antineutrophilic cytoplasmatic antibody (ANCA)-associated vasculitis given weight loss, lack of appetite, malaise, MRI findings and hypertension), mitochondrial antibody immunoglobulin G (obtained in consideration of primary biliary cholangitis given abdominal discomfort and elevated liver enzymes), rheumatoid factor (presumably obtained for broad investigation of autoimmune pathology given multi-system involvement), mycoplasma pneumonia antibodies (obtained in consideration of mycoplasma pneumonia given concern for a recent cold and extrapulmonary features such as gastrointestinal symptoms), hepatitis panel (to investigate elevated liver enzymes), antinuclear antibody (for investigation of autoimmune etiology), and anti-double-stranded deoxyribonucleic acid antibody (obtained in consideration of systemic lupus erythematosus given hypertension, weight loss, and elevated inflammatory markers). The pediatric autoimmune encephalitis cerebrospinal fluid (CSF) and serum were negative, as well as the meningitis/encephalitis panel profiles (all obtained due to altered mental status and MRI and EEG abnormalities). CSF protein and cell count were within normal limits, but glucose was elevated to 85 mg/dL (range 45–80), and CSF was positive for oligoclonal bands.

During the first several days of hospitalization, the patient struggled to eat the foods available on the hospital menu, although she would occasionally eat food brought by her parents, which raised concern for selective eating involving safety foods. As her nausea and vomiting improved with antiemetics and fluids but her nutritional status remained poor, psychiatry recommended that the patient be placed on a modified eating disorder protocol on hospital day 8. The psychiatry team did not want to implement a full eating disorder protocol solely based on the patient history and recent weight loss since there were symptoms concerning for an underlying medical etiology, but they felt that it was time to initiate an eating protocol since the patient was physically tolerating oral intake, needed nourishment, and was not consuming adequate nutrition on her own. The patient was not able to follow this protocol, so on hospital day 9, a nasogastric tube was placed and total parental nutrition (TPN) was initiated. The patient did not tolerate the nasogastric tube due to anxiety and vomiting, and she remained on TPN for 7 days until oral intake improved with the support of a dietician.

On hospital day 4, nephrology was consulted for hypertension of about 150/90 mm Hg and recommended investigation for porphyria due to abdominal symptoms, cola-colored urine, and persistent mild hyponatremia. They also expressed concern for vasculitis given multi-organ involvement, including the brain MRI findings. On hospital day 9, results for random urine porphyria analyses revealed a porphobilinogen (PBG) elevated to 314.5 µmol/L (range 0.0–8.8), a PBG to creatinine (CRT) ratio of 109.3 mmol/mol CRT (range 0–0.2), a uroporphyrin to CRT ratio of 87 µmol/mol CRT (range 0–4), a hepatocarboxyporphyrin to CRT ratio of 6 µmol/mol CRT (range 0–2), a coproporphyrin I to CRT ratio of 10 µmol/mol CRT (range 0–6), a coproporphyrin III to CRT ratio of 82 µmol/mol CRT (range 0–14), aminolevulinic acid (ALA) of 158 µmol/L (range 0–35), and ALA to CRT ratio of 94.1 mg/g CRT (range 1.5–5.3). The laboratory assays utilized to detect porphyrias involve measurement of the urinary excretion of intermediates of the heme biosynthesis pathway. Levels of porphyrin precursors are then normalized to CRT concentration and reported as a ratio to improve the accuracy of results. Total plasma/serum porphyrins were elevated to 36 nmol/L (range 0–15). Intravenous (IV) hemin 4 mg/kg was started on hospital day 10 for 4 days to treat suspected AIP. Levels of porphyrin precursors are elevated during acute porphyria neurovisceral attacks but may return to normal during asymptomatic periods of the disease. Diagnosis was later confirmed with genetic testing that revealed that the patient was heterozygous for a pathogenic variant (c.510_511del; p.Asn171Hisfs*⁣*^*∗*^38) of the hydroxymethylbilane synthase (*HMBS*) gene. Throughout treatment, the mental status of the patient steadily improved and stabilized so that she returned to neurologic baseline. Her thoughts were consistently logical, she remembered conversations with various team members, and her attention and orientation did not fluctuate. She remained guarded and irritable during discussions that involved food intake and body image. She frequently voiced concerns about high anxiety that had been chronic but was heightened by the acute stressors of illness and hospitalization.

The patient was ultimately discharged on hospital day 17 after completing hemin treatment and tolerating oral intake increasingly well. She was discharged on escitalopram 5 mg daily for anxiety, amlodipine 5 mg twice daily for hypertension, levetiracetam 500 mg twice daily for seizures, and intranasal midazolam 5 mg as a rescue medication for seizures, although she did not have any additional seizures after admission. Follow-up outpatient appointments were scheduled with primary care, metabolics, psychiatry, and nephrology. A timeline of hospital events is included for reference in the supporting information section.

## 3. Discussion

Although the diagnostic challenges that surround AIP due to its broad range of symptoms and rarity (estimated prevalence of symptomatic AIP is 1 in 170,000) have been discussed in the literature, there is a paucity of information about the interplay between AIP and eating disorders [5]. In one case study that emphasizes the vicious cycle between AIP and untreated anorexia, the patient had a clear anorexia nervosa diagnosis that precipitated his porphyria attack [6]. In AIP, the impaired function of PBG-deaminase, the third enzyme in heme biosynthesis, forms a bottleneck in the pathway that produces heme, so that any trigger that causes an increase in the demand for heme leads to toxic accumulation of the intermediates that precede the bottleneck, ALA and PBG [7]. Caloric restriction is one of the known triggers for porphyria attacks because it increases the demand for heme and upregulates the first enzyme in heme biosynthesis, ALA synthase-1 (ALAS1), which thus leads to a buildup of ALA and PBG since these precursors cannot be adequately utilized by the defective PBG-deaminase. In fact, prior to the introduction of therapeutic heme, carbohydrate loading was the standard treatment for AIP attacks, and it is still recommended in mild attacks or when heme is not available [8]. Conversely, at least two other cases support the possibility that AIP may lead to the development of anorexia nervosa, perhaps related to the weight fluctuations that accompany this episodic illness that involves loss of appetite and nausea [6]. Because caloric restriction and fasting precipitate AIP attacks, it is especially important for AIP patients to foster a healthy relationship with food. Furthermore, AIP has been associated in some studies with several psychiatric diagnoses, including depression, bipolar disorder, and schizophrenia, which underscores the impact that high levels of porphyrin precursors have on the brain [7, 9]. More research is needed to further investigate the connection between AIP and psychiatric disorders since a large cohort study did not find an association between AIP and the main psychiatric diagnostic categories (mood disorders, psychosis, addiction, and neurotic disorders), although a difference in relative risk was noted between the reference group and psychiatric disease overall as well as behavioral disorders [10].

In this case, there was strong concern for anorexia nervosa given the patient's history of caloric restriction and purging, chronic weight loss that the patient and family underestimated, and current food selectivity and discomfort discussing body image and food intake. Anorexia may have clouded the diagnosis initially because seizures may result from electrolyte abnormalities associated with eating disorders, including hyponatremia, which was noted in this patient. The dark urine color of the patient, mildly elevated liver enzymes, and urine ketones could be explained by severe dehydration and a malnourished state related to disordered eating. Furthermore, it is not uncommon for severely malnourished patients to present with mental status changes that include poor attention span and thought abnormalities. However, given that additional studies such as MRI demonstrated positive findings, it became clear that anorexia nervosa alone did not account for the full presentation.

Additionally, we found no literature that directly elucidates the impact of increasingly common cannabis use on the diagnosis of AIP. A recent case report describes the challenges that delayed the diagnosis of AIP in a 30-year-old woman who ultimately required admission to the intensive care unit [11]. During one of her emergency department presentations, her vomiting was suspected to be related to an increase in cannabis use. In our case, a differential diagnosis of cannabinoid hyperemesis syndrome vs viral gastroenteritis was given in the emergency department, which highlights the importance of viewing cannabinoid hyperemesis syndrome as a diagnosis of exclusion rather than a primary explanation for severe vomiting, especially because patients sometimes use cannabis to mitigate nausea. Notably, the patient was initially discharged from the emergency department with a prescription for ondansetron, and cannabinoid hyperemesis syndrome is classically resistant to mainstay antiemetics. Both cannabinoid hyperemesis syndrome and AIP symptoms would likely not resolve without addressing the underlying pathology by discontinuing all cannabis use or treating with glucose and heme, respectively. These cases underscore that premature and ill-founded attribution of gastrointestinal symptoms to cannabis use or cannabinoid hyperemesis syndrome leads to avoidance of a thorough medical workup on initial presentation and ultimately delays diagnosis.

Several sources recommend cessation from substances such as alcohol and cannabis as part of the treatment for AIP, but there is no clear evidence that cannabis itself exacerbates AIP like alcohol does, although a combination of cannabis and alcohol has been associated with recurrent attacks [12, 13]. Notably, animal research examining repeated administration of the cannabinoid CB_1_ receptor agonist CP-55,940 on heme metabolism found that treatment with CP-55,940 actually decreased hepatic activities of ALAS1, which indicates that cannabis may be a candidate for future research as a safe analgesic remedy in AIP [14]. It is important to consider that long-term cannabis use may exacerbate depressive symptoms, which are a psychiatric manifestation of AIP, and may increase the risk of adolescents developing depression and suicidal behavior later in life [15–18].

Several early findings in this case prompted further work-up beyond suspicion for an eating disorder and cannabinoid hyperemesis syndrome. After several days of hospitalization, even after the patient was no longer actively vomiting, her blood pressure remained elevated. Her electrolytes were not abnormal enough to account for seizures, and her altered mental status was abrupt. The MRI was consistent with posterior reversible encephalopathy syndrome (PRES), which represents a neurotoxic state caused by blood–brain barrier disruption and is linked with AIP [19]. The EEG results supported encephalopathy, though they may have been skewed by benzodiazepine administration while the patient was in the emergency department. The CSF finding of oligoclonal bands represents a neurologic pathology but has not yet been associated with AIP. Ultimately, a diagnosis of AIP is easily confirmed by obtaining urine porphyria levels, but these tests are not routinely ordered, and thus a high index of suspicion is required. Since AIP is an autosomal dominant disorder, family history may aid in diagnosis in some cases, but due to the low penetrance of the disease, most carriers are asymptomatic. In this case, there was no known family history of AIP, and it was only after the patient received her diagnosis that it was discovered that her father was an asymptomatic carrier of the AIP gene variant.

The key to timely and accurate diagnosis of AIP in this case was early collaboration among multiple specialties, with nephrology ultimately requesting porphyria labs. On hospital day 2, the primary medicine team consulted psychiatry, neurology, and nutrition; on hospital day 4, nephrology was consulted; on hospital day 5, gastroenterology was consulted; on hospital day 9, hematology/oncology and genetics were consulted; and on hospital day 15, neurosurgery was consulted. Effective management of this patient after hospital discharge will involve integration of these specialty recommendations with an emphasis on adequate nutrition as the patient follows along with psychiatry.

## 4. Conclusion

This case represents a unique diagnostic crux in which attributing presenting symptoms to elements of the patient's history delayed the primary diagnosis of AIP. Suspicion should be high for an AIP attack when there is unexplained severe vomiting and abdominal pain, particularly if hypertension, hyponatremia, and constipation are also present. Mental status changes, seizures, and muscle weakness further increase concern for AIP. Red or brown urine is consistent with AIP but is not always present, even during attacks [20]. Once a diagnosis of AIP is made, it is vital to perform a full psychosocial assessment that evaluates dietary habits and substance use, since these characteristics could increase the risk for future attacks, as well as any psychiatric conditions, since psychiatric manifestations of AIP are broad and more common than in the general population.

## Data Availability

The data that support the findings of this study are available from the corresponding author upon reasonable request.

## Supporting Information

Supporting Information We have included a timeline of relevant hospital events that summarizes any important symptoms, tests, consultations, or actions taken on each hospital day.

## References

[1] Sorensen C. J., DeSanto K., Borgelt L., Phillips K. T., Monte A. A. (2017). Cannabinoid Hyperemesis Syndrome: Diagnosis, Pathophysiology, and Treatment—A Systematic Review. *Journal of Medical Toxicology*.

[2] Klump K. L., Miller K. B., Keel P. K., McGue M., Iacono W. G. (2001). Genetic and Environmental Influences on Anorexia Nervosa Syndromes in a Population-Based Twin Sample. *Psychological Medicine*.

[3] Spiritos Z., Salvador S., Mosquera D., Wilder J. (2019). Acute Intermittent Porphyria: Current Perspectives And Case Presentation. *Therapeutics and Clinical Risk Management*.

[4] Chu F., Cascella M. (2023). *Cannabinoid Hyperemesis Syndrome*.

[5] Elder G., Harper P., Badminton M., Sandberg S., Deybach J.-C. (2013). The Incidence of Inherited Porphyrias in Europe. *Journal of Inherited Metabolic Disease*.

[6] Martins C. R., Bandeira B. E. S., Martinez A. R. M., Dalgalarrondo P., Franca M. C. (2014). Porphyria and Anorexia: Cause and Effect. *Oxford Medical Case Reports*.

[7] Gonzalez-Mosquera L. F., Sonthalia S. (2023). *Acute Intermittent Porphyria*.

[8] Zhao L., Wang X., Zhang X. (2020). Therapeutic Strategies for Acute Intermittent Porphyria. *Intractable & Rare Diseases Research*.

[9] Cederlöf M., Bergen S. E., Larsson H., Landén M., Lichtenstein P. (2015). Acute Intermittent Porphyria: Comorbidity and Shared Familial Risks With Schizophrenia and Bipolar Disorder in Sweden. *British Journal of Psychiatry*.

[10] Lissing M., Vassiliou D., Floderus Y. (2023). Risk for Incident Comorbidities, Nonhepatic Cancer and Mortality in Acute Hepatic Porphyria: A Matched Cohort Study in 1244 individuals. *Journal of Inherited Metabolic Disease*.

[11] de Larrinaga Romero I., Massa B. E. L., Rodríguez-Ruiz E., Pérez-Torres D., Martínez-Martínez M., Schaller S. J. (2023). Acute Intermittent Porphyria: A Challenging Diagnosis and Treatment. *2022 Clinical Cases in Intensive Care Medicine*.

[12] Hift R. J., Meissner P. N. (2005). An Analysis of 112 Acute Porphyric Attacks in Cape Town, South Africa: Evidence that Acute Intermittent Porphyria and Variegate Porphyria Differ in Susceptibility and Severity. *Medicine*.

[13] Amador A. P., Cordero A. S., Risco P. S. (2022). Acute Intermittent Porphyria: Is Oseltamivir Safe in These Patients?. *Clinical Medicine*.

[14] Fontanellas A., Manzanares J., Garcia-Bravo M. (2005). Effects of Repeated Administration With CP-55,940, A Cannabinoid CB1 Receptor Agonist on the Metabolism of the Hepatic Heme. *The International Journal of Biochemistry & Cell Biology*.

[15] Lev-Ran S., Roerecke M., Foll B. L., George T. P., McKenzie K., Rehm J. (2014). The Association Between Cannabis use and Depression: A Systematic Review and Meta-Analysis of Longitudinal Studies, Psychol. *Psychological Medicine*.

[16] Degenhardt L., Hall W., Lynskey M. (2003). Exploring the Association Between Cannabis Use and Depression. *Addiction*.

[17] Pacheco-Colón I., Ramirez A. R., Gonzalez R. (2019). Effects of Adolescent Cannabis Use on Motivation and Depression: A Systematic Review. *Current Addiction Reports*.

[18] Gobbi G., Atkin T., Zytynski T. (2019). Association of Cannabis Use in Adolescence and Risk of Depression, Anxiety, and Suicidality in Young Adulthood. *JAMA Psychiatry*.

[19] Pischik E., Kauppinen R. (2009). Neurological Manifestations of Acute Intermittent Porphyria. *Cellular and Molecular Biology*.

[20] Whatley S. D., Badminton M. N. (2019). Acute Intermittent Porphyria. *GeneReviews*.

