# Supplementary material for: An Adolescent Female With Disordered Eating and Cannabis Use Found to Have Acute Intermittent Porphyria
Source: Case Rep Psychiatry. 2025 Jun 25;2025:8875138. doi: 10.1155/crps/8875138 (PMC12221547; doi:10.1155/crps/8875138)
Supplement: Supporting Information — We have included a timeline of relevant hospital events that summarizes any important symptoms, tests, consultations, or actions taken on each hospital day. [file 8875138.f1.docx]

| Pre-Hospital Day | **Symptoms**: Initial Presentation to the emergency department with abdominal pain and vomiting |
| --- | --- |
|  | **Actions**: Discharge to home |
| Hospital Day 1 | **Symptoms**: Return to emergency department after seizure, agitation |
|  | **Tests**: MRI brain concerning for vasculitis or encephalitis but cannot rule out hypertensive encephalopathy or posterior reversible encephalopathy syndrome (PRES) |
|  | **Actions**: Admission, intravenous (IV) fluids, antiemetics, IV steroids (x5 days) |
|  | Given haloperidol, lorazepam, and diphenhydramine for agitation |
|  | Received IV loading dose of levetiracetam, which was continued as maintenance |
| Hospital Day 2 | **Symptoms**: Resolution of emesis but continued abdominal discomfort |
|  | **Tests**: Lumbar puncture positive for oligoclonal bands and elevated glucose |
|  | EEG supporting encephalopathy |
|  | **Consults**: Psychiatry, Neurology, Nutrition |
|  | **Actions**: Continue haloperidol and lorazepam as needed for nausea and vomiting |
| Hospital Day 3 | **Tests**: Abdominal and pelvic ultrasound notable for asymmetrically prominent left renal size, gallbladder sludge, urinary bladder debris |
|  | MR Angio head-normal |
|  | **Actions**: Start IVIG (intravenous immunoglobulin) x3 days |
| Hospital Day 4 | **Tests**: Abdominal X-ray showing large and small bowel gaseous distention but no obstruction |
|  | Renal ultrasound with doppler showing no renal abnormalities |
|  | **Consults**: Nephrology |
|  | **Actions**: Start hydralazine 10 mg IV as needed for systolic blood pressure >140 mm Hg  Start 0.1 mg clonidine patch weekly |
|  | Start cyproheptadine 4 mg twice daily and calorie count with Boost available |
| Hospital Day 5 | **Consults**: Gastroenterology |
|  | **Actions**: Start amlodipine 5 mg daily |
|  | Increase omeprazole to 40 mg daily |
| Hospital Day 6 | **Symptoms**: Parents noted improvement in patient interactions but continued mild cognitive deficits (e.g., unable to spell WORLD backward, required a clue to complete delayed recall of 3 items) |
| Hospital Day 7 | **Symptoms**: Improvement in abdominal pain and improved appetite, but poor oral intake |
| Hospital Day 8 | **Symptoms**: Started modified eating disorder protocol |
| Hospital Day 9 | **Consults**: Hematology/Oncology and Genetics |
|  | **Actions**: Nasogastric tube placed (and removed) Start hyoscyamine 0.375 mg twice daily |
|  | Start total parenteral nutrition (TPN) |
| Hospital Day 10 | **Symptoms**: Recurrence of abdominal pain and emesis with 7 episodes in 24 hours |
|  | **Actions**: Start IV hemin 4 mg/kg |
| Hospital Day 12 | **Symptoms**: Improvement in abdominal pain |
| Hospital Day 13 | **Actions**: Completed hemin treatment |
|  | Start escitalopram 5 mg daily for anxiety |
|  | Start hydroxyzine 25 mg every 6 hours as needed for anxiety |
|  | Start trazodone 50 mg nightly as needed for insomnia |
| Hospital Day 14 | **Symptoms**: Resolution of abdominal pain, no further episodes of emesis |
| Hospital Day 15 | **Symptoms**: Gradually improving oral intake |
|  | **Tests**: Repeat MRI brain correlating with PRES and interval development of thin supratentorial and infratentorial subdural hemorrhages |
|  | **Consults**: Neurosurgery |
| Hospital Day 16 | **Tests**: Repeat rapid MRI brain showing no progression of previous findings |
|  | **Actions**: Discontinued TPN |
| Hospital Day 17 | **Symptoms**: Mental status examination revealed a return to baseline with demonstration of skills that had been problematic for the patient on admission (e.g., clock drawing, spelling WORLD forward and backward, serial 7s, delayed recall, and orientation) |
|  | **Actions**: Discharge to home |

Timeline of relevant hospital events.
